# Reversible Atlantic overturning despite continued Greenland ice sheet melt

**DOI:** 10.1126/sciadv.aee5373

**Published:** 2026-10-14

**Authors:** Chuncheng Guo, Shuting Yang, Ilana Schiller-Weiss, Jorge Bernales, Steffen M. Olsen, Torben Koenigk, Rashed Mahmood, Tian Tian, Klaus Wyser

**Affiliations:** ^1^ Danish Meteorological Institute, Copenhagen, Denmark.; ^2^ Department of Environmental Science, Aarhus University, Roskilde, Denmark.; ^3^ Swedish Meteorological and Hydrological Institute, Norrköping, Sweden.; ^4^ Bolin Centre for Climate Research, Stockholm University, Stockholm, Sweden.

## Abstract

The Atlantic Meridional Overturning Circulation (AMOC) is considered vulnerable to irreversible weakening or collapse under global warming and related Greenland ice sheet (GrIS) melt, yet its resilience to either forcing remains poorly constrained. Here, we use a carbon dioxide emission–driven Earth system model with an interactive GrIS to assess AMOC reversibility under a range of idealized carbon dioxide emission pathways that produce near-linear global warming up to 10 kelvins (K), stabilization across 1.5 to 9 K, and subsequent cooling. We find that although the AMOC reaches threshold-defined collapsed states, these do not represent dynamical tipping in this model: The overturning weakens quasi-linearly with global mean temperature increase yet consistently and promptly recovers under cooling. In contrast, GrIS mass loss accelerates with warming, continues through stabilizations, and is only slowed by cooling, committing the planet to long-term sea level rise. These results reveal contrasting resilience across climate system components: Under transient carbon dioxide forcing, AMOC strength remains dynamically reversible despite continued Greenland meltwater input, whereas the GrIS is locked into persistent decline. Our findings underscore the urgency of rapid emission cuts to limit climate overshoot, AMOC weakening, and irreversible ice sheet loss.

## INTRODUCTION

The Atlantic Meridional Overturning Circulation (AMOC) redistributes heat and salt globally by carrying warm surface waters northward and returning cold deep waters southward (*1*, *2*). A major weakening of the AMOC can severely affect global and especially northwestern European climate and weather patterns, including regional temperature, precipitation, and sea level (*3*–*8*).

AMOC observations from the RAPID array at 26.5°N since the early 2000s reveal large interannual variability with only a marginally significant negative trend, and the observational record remains too short to robustly separate any forced trend from natural variabilities (*9*). Proxy-based and indirect reconstructions provide a longer perspective on the evolution of the AMOC over the historical period, although their interpretation remains contested [e.g., (*10*, *11*)]. Whether a significant anthropogenically driven AMOC trend has emerged over the past century remains unclear (*12*).

Under continued warming, enhanced surface heating, precipitation, and Greenland ice sheet (GrIS) meltwater are projected to strengthen stratification in the subpolar North Atlantic, suppressing deep-water formation and thereby weakening the AMOC (*13*, *14*). A weakening AMOC reduces its own northward salt transport, which may reinforce further decline through the salt-advection feedback (*15*).

Analytical and numerical studies (mainly based on freshwater forcing) indicate that the AMOC may be bistable, such that a weakening or “collapse” could be irreversible for long periods even under reduced forcing (*15*–*18*). Throughout this manuscript, we distinguish two meanings of AMOC collapse: (i) a threshold-defined collapse (denoted “collapse” hereafter), in which AMOC strength falls below a certain criterion, such as 6 Sverdrup (Sv; 1 Sv = 10^6^ m^3^/s) at a fixed depth or an 80% reduction relative to preindustrial (PI) values at variable depth, both at 26° N (*19*, *20*); and (ii) a dynamical collapse or tipping (denoted tipping hereafter), referring to a potentially irreversible, and often abrupt, transition associated with the bistability framework. Evidence of AMOC bistability comes from multiple lines: box model theory (*15*), paleoclimate reconstructions [e.g., (*21*)], and freshwater hosing experiments in coupled climate models [e.g., (*18*)]. Statistical analyses of proxy records of AMOC strength and model-derived physics-based early-warning indicators have raised the possibility of approaching a tipping point (*18*, *22*, *23*), although these signals remain debated and the proxies contested (*24*, *25*).

Climate models participating in the Coupled Model Intercomparison Project (CMIP), typically not accounting for GrIS mass loss, consistently project continued weakening of the AMOC through the 21st century, although the magnitude and timing vary widely across models and main SSP scenarios (*26*–*28*). Physically based emergent constraints of the CMIP6 experiments yield conflicting estimates, suggesting either limited or stronger weakening before the end of the century compared to unconstrained results, depending on the observable variables and methods used (*29*, *30*). Beyond 2100, model projections indicate a risk of a continued evolution toward a very weak, threshold-defined “collapsed” AMOC state, e.g., a stream function weaker than 6 Sv at ∼1000 m depth and 26°N, under extended SSP5-8.5 scenario with strong global warming (*19*); even in low-emission pathways such as extended SSP1-2.6, a “collapse” cannot be entirely ruled out, although the risk is substantially reduced. Under the mid-emission extended SSP2-4.5 scenario, one model (of two, both belonging to the same model family) exhibits bifurcation behavior, with ensemble members diverging between continuation to a “collapsed” state and recovery, consistent with a noise-induced transition (*31*). Similar bifurcation behavior was also found at certain fixed CO_2_ levels in idealized simulations (*32*).

However, whether these “collapsed” states represent genuine dynamical tipping in a warming future remains unclear, as most climate model studies consider either warming alone without reversed forcing (i.e., cooling) (*19*, *33*) or idealized freshwater perturbations (*18*, *34*). This leaves unresolved whether warming-driven AMOC weakening or “collapse” is reversible. A few recent studies examined AMOC reversibility under temperature stabilization or overshoot (*35*, *36*) and generally found that AMOC can recover once temperatures stabilize or decrease at 21st century–relevant warming levels. However, most models in these studies do not reach a “collapsed” state before cooling is applied, and none of these models include an interactive GrIS providing physically consistent, time-varying meltwater forcing.

Understanding how the AMOC behaves under realistic, time-varying GrIS forcing and how the GrIS itself responds to warming and cooling in a coupled setting where AMOC-driven heat transport also shapes Greenland climate and outlet glaciers (*37*) is essential for assessing Earth system resilience. The GrIS itself represents a potential tipping element whose response to changing climate conditions is governed by slow elevation adjustment and dynamical feedback processes (*38*). As long as global temperatures remain above PI levels, GrIS mass loss will continue, at a rate that depends on the level of warming reached; even present-day warming may already commit it to substantial long-term loss (*39*). The eventual magnitude of that loss, however, still depends on the warming trajectory that follows: Temperature reductions after a period of overshoot can substantially limit the loss (*40*). Despite projections of strongly increasing freshwater input from GrIS melt (*41*, *42*), many Earth system models (ESMs) used in AMOC studies either replace potential feedback mechanisms between climate and ice sheets by highly idealized surrogates or exclude GrIS melting entirely—an omission that might underestimate future AMOC weakening (*43*, *44*).

Here, we assess AMOC stability and reversibility under global warming and dynamical GrIS melt using EC-Earth3-ESM-1 (see Materials and Methods), one of the few emission-driven ESMs with an interactive GrIS that produces realistic meltwater fluxes in recent decades. We do this under idealized CO_2_ emission pathways, following and extending the ESM experiment protocol of the international Tipping Points Modeling Intercomparison Project (TIPMIP) (*45*, *46*). We deliberately span a wide range of global warming levels (GWLs; 1.5 to 9 K), including very high values beyond typical 21st century projections, to probe the limits of AMOC stability and to search for a potential tipping point. Each GWL includes near-symmetric warming, stabilization, and cooling phases with positive, zero, and negative CO_2_ emissions, respectively (see Materials and Methods). By combining this transient forcing framework with a high-complexity ESM that directly links emissions to temperature and climate responses (*47*), we show that although the AMOC attains threshold-defined “collapsed” states at high warming levels, these states remain dynamically reversible. This is in stark contrast to the persistent decline of the GrIS, highlighting contrasting resilience across climate system components.

## RESULTS

### Reversible AMOC weakening under CO_2_ forcing

Our emission-driven transient forcing experiments, by design (see Materials and Methods), produce a linear global mean warming of 0.2 K/decade up to about 10 K (Fig. 1A). This is followed by a zero-emission phase at prescribed GWLs (1.5 to 9 K)—hereafter referred to as stabilization, although temperatures do not fully stabilize at very high GWLs (see below). Constant negative emissions are then applied after 50 years of stabilization, initiating global cooling. The cooling rate is comparable in magnitude but slightly faster than the warming rate (for example, −0.25 K/decade in the 3- to 5-K ramp-downs).

**Fig. 1.**
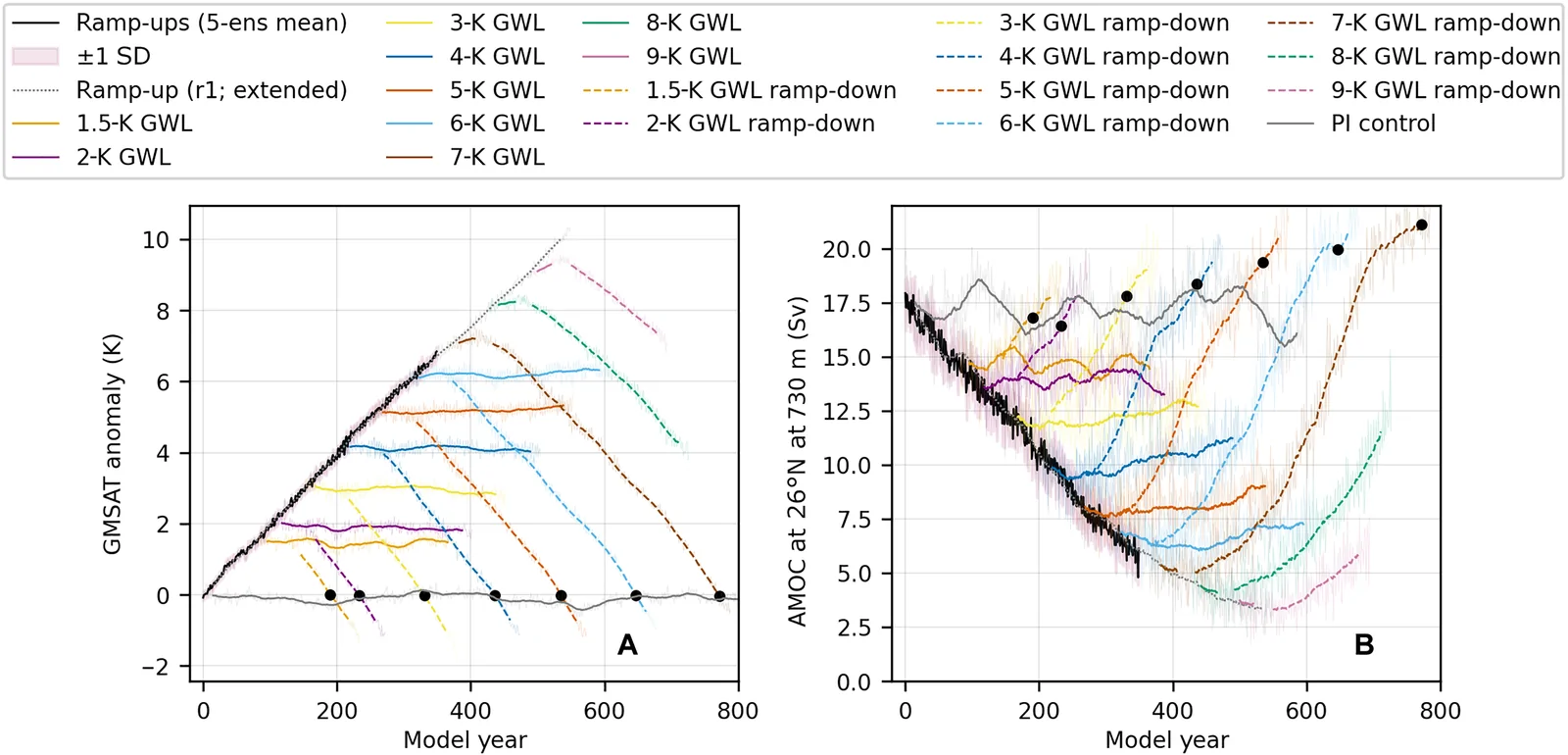
Evolution of GMSAT and the strength of AMOC at 26°N under idealized CO_2_ emission scenarios. (**A**) GMSAT [in kelvin (K)] anomaly relative to the PI in the ramp-up (positive emissions), stabilization (zero emissions), and ramp-down (negative emissions) experiments with EC-Earth3-ESM-1. Global warming follows the imposed rate of 0.2 K/decade, stabilizes around the prescribed GWLs, and declines nearly symmetrically (albeit slightly faster) during ramp-down. (**B**) The corresponding AMOC strength at 26°N (defined at the depth of the PI maximum, 730 m; see Materials and Methods). In both panels, the black line and light red shadings indicate five-member ensemble mean and one SD of the annual means in the ramp-up runs; the gray dotted line indicates one extended ramp-up run; in the PI (gray solid lines), stabilization (colored solid lines), and ramp-down (colored dashed lines) runs, thin lines and thick lines are annual means and 31-year running means, respectively. Black dots indicate the points when the GMSATs return to the PI level in the ramp-down experiments. The AMOC weakens quasi-linearly with global warming, stabilizes as temperature stabilizes, and recovers during cooling.

The warming levels explored here span and extend beyond 21st century CMIP6 projections. For reference, the high-emission scenario SSP5-8.5 yields a likely range of 3.3 to 5.7 K by 2100 (*28*). We therefore refer to 1.5 to 6 K as 21st century–relevant GWLs and 7 to 9 K as very high GWLs, the latter addressing multicentennial timescales and pathways with no direct policy relevance for the 21st century. These extended very high GWLs enable us to assess whether AMOC can “collapse” under a wide range of warming and whether it can be reversed.

The AMOC strength is defined here as the overturning stream function at 26°N at a fixed depth of 730 m (see Discussion and Materials and Methods for the robustness of the results to the choice of depth and latitude). In the PI control experiment, AMOC strength exhibits multidecadal variability around a mean of about 17 Sv (Fig. 1B). During the ramp-up, the AMOC weakens quasi-linearly with the increase in global mean surface air temperature (GMSAT) at roughly −1.6 Sv per degree of warming (fig. S1). Across all five ensemble members, AMOC evolution is highly consistent and declines to about 6 Sv at 7-K warming, showing no “collapse” within 21st century–relevant GWLs. Additional sensitivity tests with doubled and tripled CO_2_ emission rates yield nearly identical AMOC trajectories during warming when compared against global mean temperature (fig. S2). This indicates that AMOC weakening depends primarily on the level of warming rather than its rate in this model (within the forcing range tested), with no evidence that more rapid forcing could trigger a nonlinear transition (*48*). These results reinforce the interpretation that AMOC weakening scales with global mean temperature.

Beyond 7 K, an extended ramp-up simulation shows that AMOC weakening continues, reaching a strength of only ∼3 Sv at 10-K warming. By certain threshold-based criteria, e.g., below 6 Sv at a fixed depth at 26°N (*19*), the simulated AMOC strength at very high warming constitutes a “collapsed” state.

Across all zero-emission runs spanning 1.5- to 6-K GWLs, AMOC weakening ceases as soon as emissions stop and temperatures stabilize, remaining stable for centuries with no or only weak trends. Variability also declines as the mean strength weakens, consistent with previous studies (*49*, *50*). At very high GWLs (7 to 9 K), AMOC continues to weaken during the short stabilization periods (50 years), responding to the continued increase in GMSAT even after emissions cease.

Once CO_2_ emissions turn negative and temperatures decrease, the AMOC undergoes a quasi-linear recovery that mirrors its prior weakening during the ramp-up phase (Fig. 1B and fig. S1). Even in the very high GWL ramp-downs, the AMOC exhibits a prompt recovery mirroring the weakening, and ultimately undergoes a vigorous rebound. “Prompt” here refers to the AMOC strength tracking the cooling closely, rather than to any fast absolute timescale: The ramp-downs themselves and the associated AMOC recovery span centuries from the very high GWLs.

As global temperature returns to the PI level, the AMOC overshoots its initial strength in the ramp-downs from higher GWLs, with overshoot magnitude increasing with peak warming (Fig. 1B and fig. S1; the 8- and 9-K ramp-down simulations do not extend to PI temperatures; see Materials and Methods). This overshoot behavior—also seen in temperature overshoot scenarios without full return to PI temperatures (*36*)—has been linked to salinity buildup in the subtropical Atlantic (*51*), which provides a source of increased upper-ocean density that invigorates deep convection.

In summary, the AMOC weakens quasi-linearly and substantially, reaching threshold-defined “collapsed” states at very high GWLs, but exhibits no abrupt or irreversible transition in this model. Thus, the AMOC does not “collapse” (or cross a tipping point) under the dynamical definition. This finding is robust to the choice of the AMOC metric, as confirmed by alternative metrics with variable depths and latitudes (see Discussion).

### Mixed-layer recovery and reorganization

Freshening from ice melt and precipitation, together with surface warming, strengthens upper-ocean stratification and suppresses deep convection in the northern North Atlantic. This self-reinforcing “convective shutdown” feedback (*52*–*54*) promotes persistent shoaling of the mixed layer and reduced deep-water formation.

In the PI control, March mixed-layer depths (MLDs; see Materials and Methods), used as an indicator of dense water formation by deep convection, are largest in the subpolar gyre (mainly in the Labrador and Irminger Seas) and northern Nordic Seas (Fig. 2A). Under increasing stabilized GWLs, mixed layers shoal progressively and asynchronously across the major convection sites (Fig. 2, B to I). The Labrador and Irminger Seas experience the strongest decline, with deep convection largely disappearing by about 3 to 4 K, whereas the Nordic Seas retain winter mixing, remaining at ∼200 m even at very high GWLs.

**Fig. 2.**
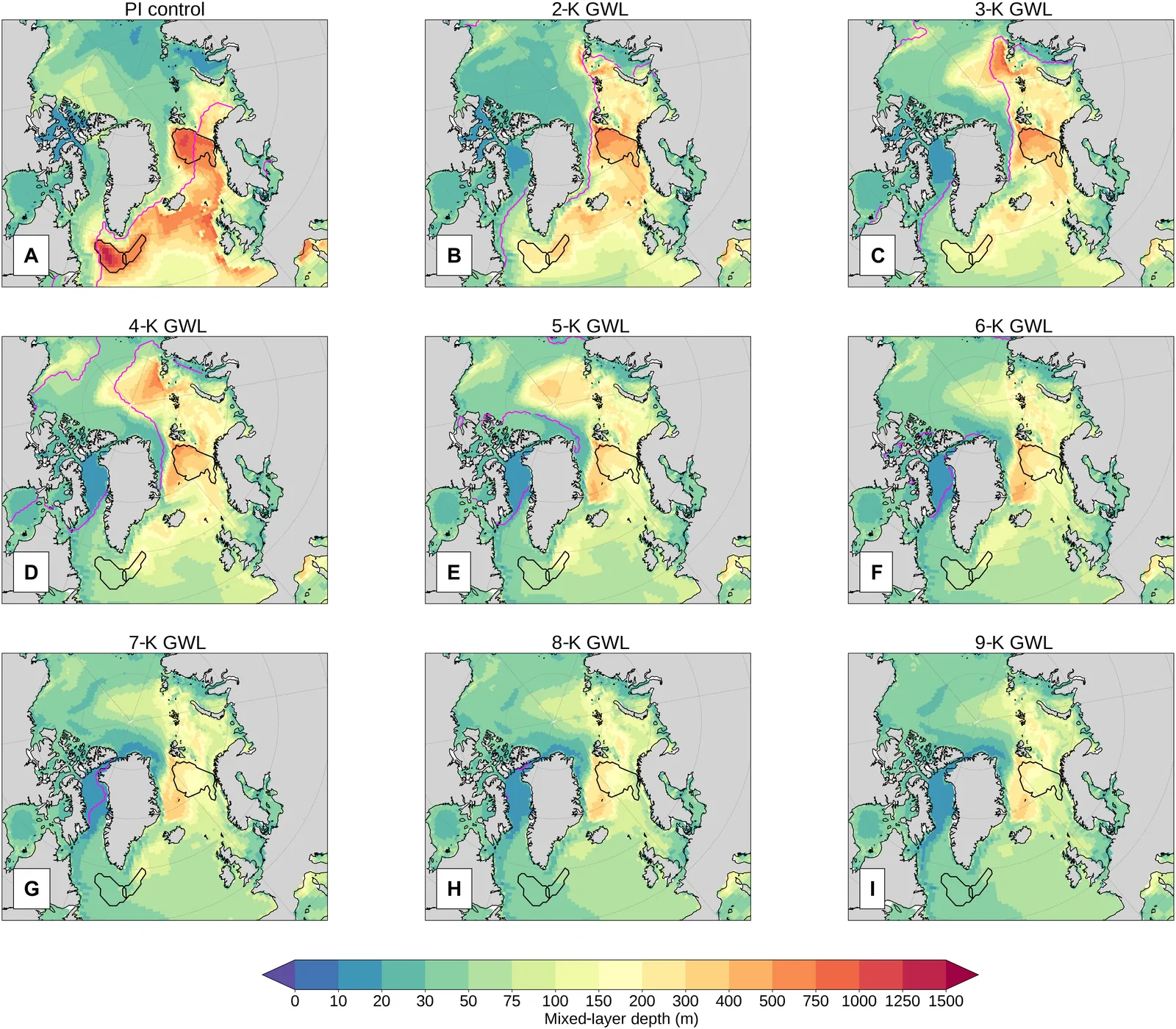
Spatial distributions of March MLDs under PI and stabilized warming conditions. Maps show March mean MLDs (in meters) averaged for the whole periods of the PI control (**A**) and GWLs of 2 to 9 K (**B** to **I**). Black contours delineate areas where the PI MLDs exceed 650 m, indicating the main deep convection sites in the Labrador, Irminger, and Nordic Seas. Magenta contours indicate the March sea ice edge, defined by the 15% sea ice concentration threshold. Shoaling of the MLDs with increasing temperatures illustrates the progressive and asynchronous suppression of deep convection in different sites under sustained warming.

Time series of March MLDs (Fig. 3) further illustrate these regional contrasts. In the Labrador and Irminger Seas, MLDs shoal rapidly during warming, e.g., a reduction of more than 90% at 4 K in the Labrador Sea, effectively indicating a cessation of deep convection. MLDs in the subpolar region remain shallow during stabilization and deepen again during cooling, with longer delays at higher GWLs. In the Nordic Seas, MLDs evolve more gradually during warming, with early shoaling, a plateau near 400 m between 1- and 4-K warming, and a further decline to about 200 m at higher GWLs—a reduction of ∼75% compared to PI in the fixed area–averaged metric (see Materials and Methods). However, main winter mixing migrates to the western Nordic Seas (Fig. 2), providing a continuing source of dense water that helps sustain the AMOC when subpolar convection ceases (*55*–*57*). Once cooling begins during ramp-downs, MLDs in the Nordic Seas deepen again without delays (Fig. 3C).

**Fig. 3.**
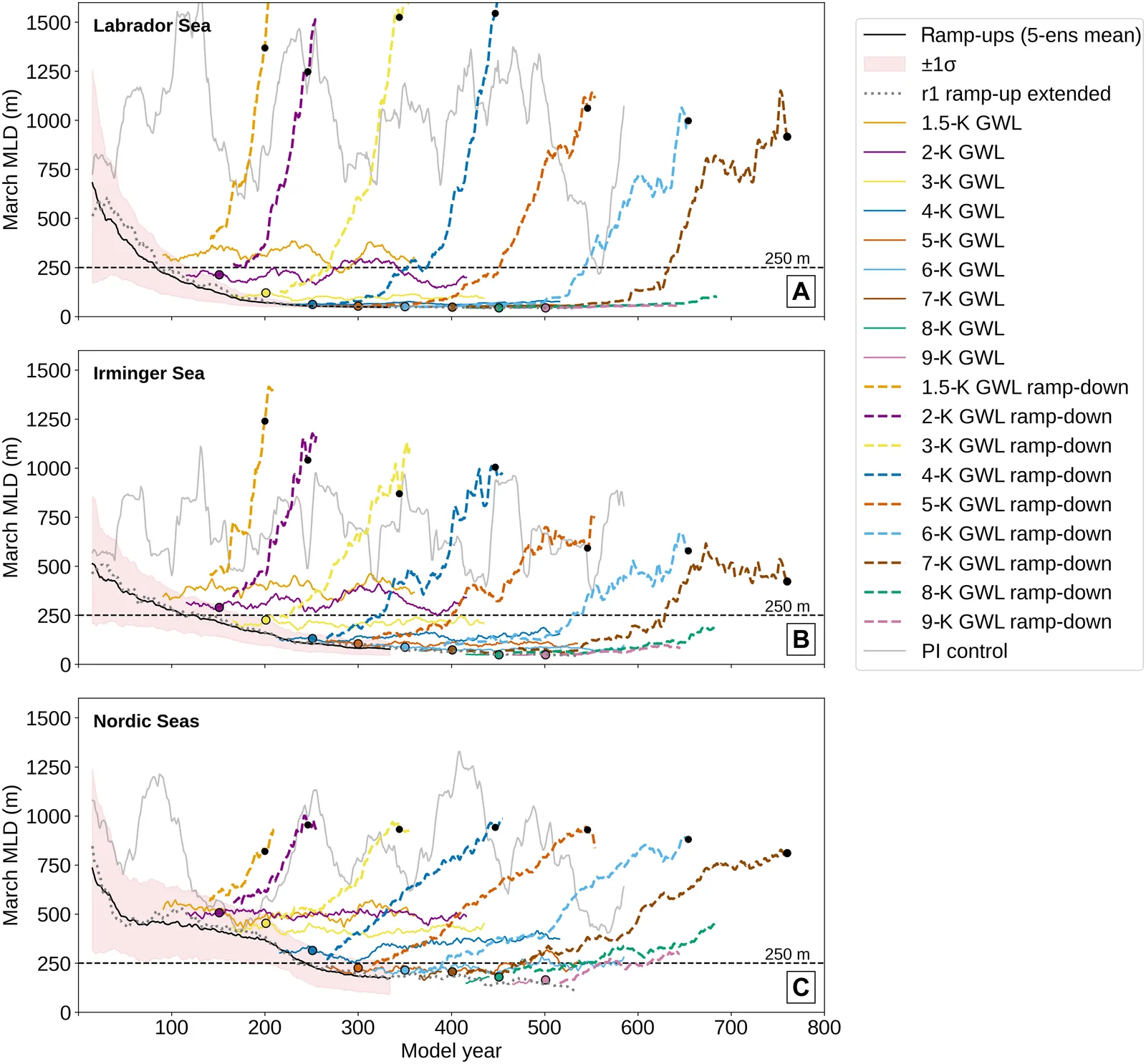
Temporal evolution of March MLD under idealized CO_2_ emission scenarios. Time series of 31-year running mean March MLDs (in meters) in the (**A**) Labrador, (**B**) Irminger, and (**C**) Nordic Seas. In all panels, the gray line shows the PI, and black/colored lines correspond to the ramp-up (warming), stabilization, and ramp-down (cooling) experiments shown in Fig. 1. Light red shadings indicate the spread of the five-member ensemble, and the gray dotted line indicates one extended ramp-up run. The black dashed line indicates the 250-m level. Colored dots mark the onset of ramp-downs 50 years after stabilization, and black dots mark when the GMSATs return to PI levels. The transition from shoaling during warming to deepening during cooling illustrates the recovery of deep convection underpinning the Atlantic overturning.

During ramp-downs, deep convection ultimately recovers in all the deep-convection regions once GMSATs return to the PI level. The spatial pattern, however, differs from the initial PI state (fig. S3). Major convection sites shift and expand—including a broader deep mixing area in the Labrador Sea consistent with the AMOC overshoot—suggesting that the AMOC recovery arises from a broader spatial reorganization of deep-water formation rather than a simple return to the initial pattern. In addition, these results indicate that recovery of AMOC strength does not imply that the wider North Atlantic system is equally reversible.

During the warm phases, new regions of deep winter mixing appear in the Eurasian Basin of the Arctic Ocean, mainly north of Svalbard and Severnaya Zemlya, closely following the winter sea ice edge (Fig. 2). These Arctic convection sites emerge around 2-K warming, peak near 3 to 4 K, and weaken again at higher GWLs. This Arctic MLD deepening and the associated formation of dense water help sustain the weakened AMOC and, likely in combination with the persistent mixing in the western Nordic Seas, also support AMOC recovery during cooling. The emergence of Arctic deep mixing is consistent with recent model evidence that future AMOC strength may increasingly depend on a poleward shift of deep-water formation as sea ice retreats (*58*, *59*), although these shifts remain uncommon in CMIP6 projections (*60*).

Overall, the mixed layer across the North Atlantic and Arctic exhibits remarkable resilience: Major deep convection sites not only recover after suppression but also reorganize spatially, with new hotspots emerging as sea ice retreats, providing sources of dense water that underpins AMOC stability and reversibility in our simulations. We test this resilience against a specific mixed layer–based criterion proposed for AMOC tipping in Discussion.

### Resilient overturning, vulnerable ice

Idealized hosing experiments show that sustained freshwater input—such as 0.3 Sv in the multimodel North Atlantic Hosing Model Intercomparison Project (NAHosMIP) (*34*)—can lead to a major AMOC weakening or “collapse”. Today, the GrIS contributes a much smaller freshwater flux to the North Atlantic, on the order of 0.01 Sv (*42*) (the conversion between ocean volume transport unit Sv and ice loss rate is 0.01 Sv ≈ 320 gigatonnes (Gt)/year). The simulated historical evolution of GrIS mass in our model is consistent with this value, although with some overestimation (e.g., ∼4100 Gt-modeled ice loss from 2002 to 2014 versus ∼3000 Gt-observed; Fig. 4C, inset).

**Fig. 4.**
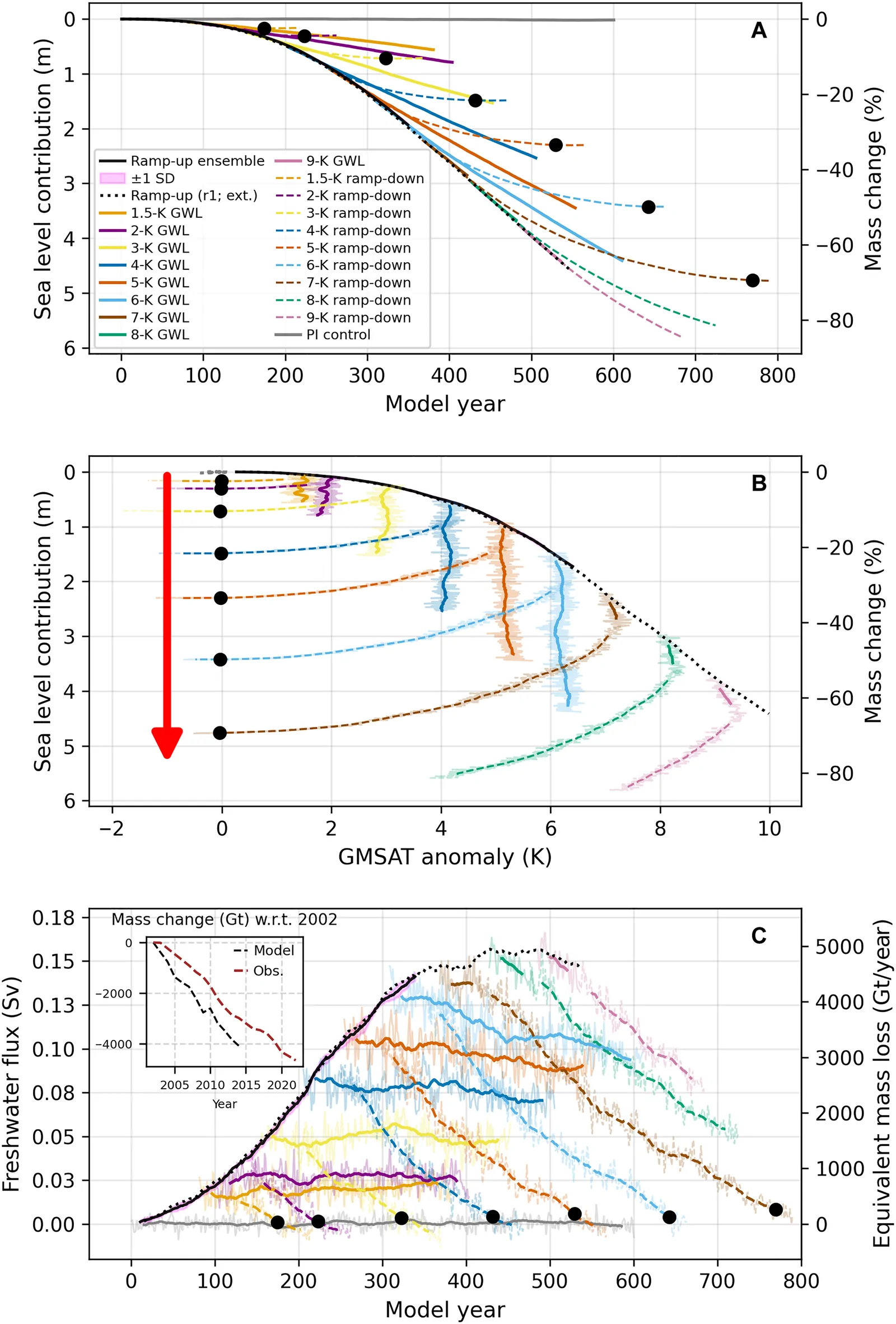
Evolution of GrIS mass and freshwater flux under idealized CO_2_ emission scenarios. Time series of GrIS mass change as percentage (right axis) and equivalent eustatic sea level rise (in meters; left axis) relative to the PI initial state, plotted against (**A**) model years and (**B**) GMSAT anomalies—the latter illustrating hysteresis (highlighted by red arrow) between warming and cooling trajectories. (**C**) Corresponding freshwater fluxes from the GrIS mass changes into the ocean (in Sv; left axis) and the equivalent mass loss rate (in Gt/year; right axis) across the full set of experiments. The conversion between ocean volume transport unit Sv and ice loss rate is 0.1 Sv ≈ 3200 Gt/year. The inset in (C) shows historical mass change (in Gt) since 2002 compared against satellite observations, demonstrating broad agreement. These are GRACE and GRACE-FO gravimetric estimates of Greenland ice mass balance (*83*, *84*). Annual uncertainties in the series are typically ±70 Gt—much smaller than the model observation discrepancy—and are therefore not shown. In all panels, the gray line shows the PI control simulation, and black/colored lines correspond to the ramp-up (warming), stabilization, and ramp-down (cooling) experiments shown in Fig. 1. The dotted line indicates one extended ramp-up run. Black dots mark when the GMSATs return to PI levels. These simulations showcase continued Greenland meltwater release and net ice mass loss during global stabilization and cooling scenarios, leading to a substantially smaller quasi-equilibrium ice sheet state even after global mean temperatures return to PI levels, in contrast to the reversible AMOC response.

Under global warming, GrIS meltwater input is expected to increase strongly (*41*, *42*), which could further suppress deep convection in the Labrador and Irminger Seas and weaken the AMOC (*61*–*63*). Across all our experiments, the GrIS undergoes sustained and substantial mass loss (Fig. 4, A and B), with an associated freshwater flux consistently remaining well below that in typical hosing experiments (see below). During the ramp-up, ice melt accelerates with air temperature increase, causing the ice sheet to lose more than 20% of its initial mass by 6-K global warming—equivalent to about 1.5 m of eustatic sea level rise. Although the rate of acceleration slows at higher GWLs, mass loss continues to increase, and by 10 K, GrIS has lost more than 60% of its initial mass (about 4.5-m sea level equivalent). During stabilization, the ice sheet remains in a persistent state of negative mass balance, with the rate of mass loss—and the associated meltwater flux—increasing nonlinearly with GWL (for example, ∼0.02 Sv at 2 K, ∼0.1 Sv at 5 K, and ∼0.15 Sv at 8 K). Cooling during ramp-down gradually slows melting and drives the ice sheet toward a new, smaller quasi-equilibrium state as temperatures return to PI levels. However, the ice sheet has already undergone substantial, irreversible loss, producing a committed sea level rise of ∼0.2 m returning from 1.5 K, ∼3.5 m from 6 K, and ∼5 m from 7 K (see black dots in Fig. 4), the latter two cases corresponding to a loss of nearly 50 and 70% of the original mass, respectively. This hysteretic response indicates that the GrIS decline is effectively irreversible on centennial timescales.

Despite the sustained freshwater forcing, the AMOC in our model shows remarkable resilience: It weakens under warming yet consistently recovers during cooling, even while meltwater fluxes remain substantial in the early phase of ramp-downs (e.g., ∼0.15 Sv at very high GWLs). Considering that the simulated meltwater fluxes during the historical period are overestimated (see above) and that this bias may carry through if it persists into the future, the AMOC recovery reported here is a conservative estimate—lower, observation-consistent fluxes would, if anything, result in less AMOC weakening and easier recovery.

To contextualize our simulated meltwater fluxes: Peak input reaches ∼0.15 Sv at very high GWLs during stabilization (Fig. 4C). This is at the lower end of the forcing applied in standard idealized hosing experiments—for example, a step increase in uniform hosing to 0.3 Sv in NAHosMIP, where AMOC “collapses” and does not recover in some models (*34*), or a gradual ramp-up to 0.1 or 0.3 Sv over ∼100 years in the TIPMIP-OCEAN protocol (*64*). At 21st century–relevant GWLs, fluxes are weaker: At 5 K, the meltwater flux reaches ∼0.1 Sv but only after ∼240 years of gradual increase—roughly 2.5 times slower than the TIPMIP-OCEAN ramp-up rate to 0.1 Sv. Our interactive GrIS-derived forcing is therefore both weaker in magnitude and more gradual than standard hosing benchmarks; the more gradual pace, in particular, gives the ocean circulation more time to adjust. This is consistent with other coupled climate–ice sheet model studies showing that AMOC weakening is already underway before substantial GrIS meltwater occurs and that its additional effect on AMOC weakening is limited (*65*–*67*), suggesting that global warming is the dominant driver. Together, these findings suggest that the freshwater forcing commonly applied in idealized hosing experiments is stronger and more abrupt than what a dynamically coupled ice sheet produces, and this helps explain why, in our model, sustained meltwater input does not prevent AMOC recovery once cooling begins.

During temperature stabilization, however, we observe little or no AMOC recovery (Fig. 1B)—in contrast to zero-emission simulations without GrIS meltwater that show partial recovery during stabilization (*35*, *36*). This difference—apart from intermodel differences—may partly reflect the effects of sustained freshwater input during stabilization in our simulations; without active cooling to drive actual recovery, the freshwater input may be sufficient to help maintain upper-ocean stratification, although isolating its precise role would require targeted sensitivity experiments.

In contrast to the AMOC, the GrIS itself behaves fundamentally differently—melting continues throughout warming and stabilization, and cooling merely slows further decline without restoring lost ice. As a result, the relationship between Greenland ice mass and global mean temperature is strongly path dependent in our modeling framework: The final ice sheet state is determined by the maximum warming reached rather than the final temperature.

This divergence highlights contrasting resilience across climate system components. While the AMOC exhibits dynamical reversibility once forcing is reversed, the GrIS response is governed by slow topographic and ice dynamic feedbacks that render its losses effectively permanent. Even when global temperatures return to PI levels, this hysteresis commits the planet to long-term sea level rise.

## DISCUSSION

### AMOC “collapse” is not necessarily AMOC tipping

In contrast to some recent studies—mainly based on statistical or dynamical indicators—warning that the AMOC is approaching a tipping point (*22*, *23*), our transient forcing ESM simulations with interactive GrIS show no evidence of either threshold-defined “collapse” or tipping within 21st century–relevant warming levels (1.5 to 6 K). Instead, the AMOC in our model weakens quasi-linearly with global mean temperature, decreasing from about 17 Sv under PI conditions to about 6 Sv at 7-K warming, stabilizes immediately once emissions cease, and recovers promptly under cooling.

Beyond 6-K warming, the AMOC enters states that satisfy certain threshold-based criteria of “collapse” (e.g., <6 Sv at a fixed depth at 26° N) (*19*). However, these weakened states remain dynamically reversible: Under reversed forcing (i.e., cooling), the AMOC returns to a strong state without abrupt or irreversible changes. Our results therefore reveal a key conceptual distinction: AMOC states that appear “collapsed” by threshold definitions are not necessarily tipped states. Whether the AMOC behaves as a tipping element under global warming thus depends critically on the definition applied, and under the dynamical definition that is most relevant for long-term climate risk, our simulations provide no evidence of AMOC tipping within the explored warming range. The presence of prompt and full reversibility across all warming and cooling trajectories indicates that any underlying AMOC tipping—if it exists in this model—lies beyond the explored warming range (up to 10 K) and the warming durations tested here or would require qualitatively different forcing, such as freshwater hosing. This is consistent with (*68*), who find warming-induced AMOC hysteresis in CCSM4 only under extreme multicentennial warming that is well beyond the forcing pathways explored here. A related caveat is that our experiments are transient rather than quasi-equilibrium, as in classical hysteresis studies: The stabilization periods (300 years for GWLs up to 6 K and 50 years at higher GWLs) may not be long enough to reveal an instability that develops on longer, quasi-equilibrium timescales. These transient trajectories, however, are the ones most relevant for real-world climate evolution.

In classical box models and hosing experiments (*15*, *18*, *34*), AMOC stability is governed primarily by the salt-advection feedback, which produces clean hysteresis and transitions into weak states when freshwater forcing is applied. Under strong CO_2_-driven warming, however, forcings include both freshwater and heat fluxes, and dominant drivers of AMOC changes (e.g., the effects of warming vs. freshening) are less clear (*68*–*71*). Beyond the salt-advection feedback, the AMOC evolves within a strongly warmed, fully coupled Earth system shaped by multiple interacting processes and feedbacks that remain incompletely understood—including changes in Atlantic precipitation and the position of the Intertropical Convergence Zone, sea ice, and surface heat flux feedbacks (*17*, *72*–*74*). Together, these interacting processes mean that AMOC behavior under warming cannot be reduced to the classical salt-advection picture alone. Consistent with this, results from NAHosMIP (*34*) show that models in which the AMOC weakens below 5 Sv at 45°N under freshwater hosing do not recover, whereas in our very high GWL experiments, the AMOC drops well below 5 Sv using the same 45°N metric (fig. S4D) yet recovers promptly once cooling begins.

Definitions matter here too: How AMOC strength is calculated can affect how results compare across studies, particularly when assessing whether the AMOC has “collapsed.” The threshold used to define AMOC “collapse” is, to some extent, arbitrary, and the community has not converged on a common definition of AMOC strength: Studies vary in the latitude used (a single value or a range), the depth (fixed or dynamically tracked), and how much of the near-surface wind-driven cell is excluded (200 m, 500 m, or not at all). This also includes studies constraining future AMOC changes (*29*, *30*) and assessing the risk of a major AMOC weakening (*19*). Results across studies are therefore not always directly comparable, and the choice of definition can affect the result. Under global warming, a fixed-depth definition can underestimate AMOC strength when the upper overturning cell shoals, and a fixed-latitude definition cannot capture the true latitudinal maximum nor any shift in its position under warming. Our sensitivity tests illustrate the magnitude of this effect directly (see Materials and Methods and fig. S4): At the weakest AMOC state, relaxing the depth constraint alone (by calculating the maximum AMOC at 26°N below 200 m) increases AMOC strength from ∼3 to ∼5 Sv, and relaxing the latitude constraint (by calculating the maximum strength across 20°N to 50°N below 200 m) further increases it to ∼6.5 Sv—a difference of 3.5 Sv between the fixed and nonfixed location of depth and latitude. Whether a value such as 6.5 Sv should itself be called “collapse” is less important than how it is calculated; what matters is that studies assessing AMOC “collapse,” including those used to constrain future projections, report the specific depth and latitude definition used and, where feasible, test sensitivity to this choice. The result that matters most for tipping, however (full recovery under reversed forcing), is robust in our simulations and is independent of the choice of definitions.

We compare our results directly with Drijfhout *et al.* (*19*), who defined AMOC strength similarly (at ∼1000-m fixed depth and 26°N) and analyzed CMIP6 SSP scenarios extended well beyond 2100, most of which are warming only. They concluded that the AMOC declines toward a “shutdown” once it crosses a threshold of 10 Sv by 2100. Our ramp-up experiments cross this threshold at 4 K, weakening further under continued warming, and reach about 3 Sv by 10 K. Under temperature stabilization at 4 K, however, AMOC strength is overall stable and even recovers slightly. However, in the corresponding ramp-downs, with forcing actively reversed rather than continued—which is actually what allows a test for tipping—the AMOC recovers fully and promptly in all cases, including from AMOC strengths well below this threshold. Derived from warming-only scenarios, the 10 Sv threshold used by Drijfhout *et al.* (*19*) is therefore neither an indicator of AMOC shutdown (or “collapse” in our terms) nor of AMOC tipping, which requires taking reversibility into account.

### Deep convection cessation is not an AMOC tipping indicator

Drijfhout *et al.* (*19*) similarly defined a cessation of deep convection—winter MLDs below 250 m within the historical deep-convection regions—and described it as a tipping point that pushes the AMOC into a decline lasting for centuries. In our experiments, however, MLDs in both the subpolar gyre and the Nordic Seas fall below this threshold before reaching 2 and 5 K, respectively (Fig. 3), well before any threshold of AMOC “collapse,” which is not reached until beyond 7 K (Fig. 1B). However, deep convection resumes in all cases, and the AMOC recovers once cooling begins. Cessation of deep convection based on this criterion is therefore neither a tipping point in itself nor a reliable indicator of AMOC “collapse,” let alone irreversible tipping.

The fixed, historically defined region used for this criterion also cannot capture the spatial reorganization of deep convection (such as a poleward migration, which would require dynamical tracking) that helps explain the AMOC’s resilience in our simulations. With increasing warming, deep convection in the Labrador and Irminger Seas weakens and nearly vanishes, while the Nordic Seas retain partial activity (with mixing migrating toward the western Nordic Seas) and new convection sites appear (mainly between 2 and 5 K) following the retreating winter sea ice edge into the Arctic Ocean. This emergence of Arctic Ocean wintertime mixing provides dense water that helps sustain a weakened but functioning AMOC at these warming levels. During cooling, the AMOC recovers promptly, despite deep convection in the subpolar gyre resuming more slowly from high GWLs, indicating that the persistence and reorganization of winter mixing in the Nordic Seas and the Arctic, rather than recovery of the subpolar convection, appear to be the critical factor enabling AMOC recovery under cooling. This holds even after the cessation of deep convection in the model. The eventual resumption of subpolar convection contributes to the full recovery and transient overshoot of the AMOC.

### Reversible AMOC, irreversible GrIS

The AMOC stabilizes and recovers despite sustained meltwater fluxes from the interactive GrIS. While isolating the precise role of the meltwater requires targeted sensitivity experiments, our simulations show that even sustained freshwater input—reaching ∼0.1 Sv at 5 K—does not prevent AMOC recovery once cooling begins. At the same time, the freshwater fluxes in our model remain weaker in magnitude and more gradual in time than those applied in standard hosing benchmarks such as in NAHosMIP (*34*), which may partly explain why AMOC recovery is not suppressed in our results. Together, this suggests that physically consistent GrIS meltwater under transient warming scenarios is unlikely, on its own, to trigger dynamical AMOC tipping in our model.

In sharp contrast to the AMOC, the GrIS itself exhibits irreversible behavior: Mass loss accelerates with warming, continues during stabilization, and cooling merely reduces the rate of decline without restoring lost ice. This hysteretic cryospheric response produces a committed sea level rise of ∼0.2 to 5 m across warming levels from 1.5 to 7 K by the time global temperatures return to the PI level. The contrast between recoverable AMOC weakening and committed GrIS mass loss reveals profoundly different resilience across climate system components.

### Robustness and limits of the results

A key novelty of our study is the use of symmetric, emission-driven ramp-up and ramp-down experiments spanning multiple GWLs, following and substantially extending the TIPMIP ESM protocol while incorporating coupled climate–ice sheet–AMOC feedbacks. This framework explicitly allows us to test AMOC reversibility and transient hysteresis along comparable warming and cooling pathways, including climate-carbon feedbacks inherent in emission-driven forcing (*47*). By systematically spanning a wide range of warming levels under a controlled warming rate, our TIPMIP ESM design fills a critical knowledge gap and reveals AMOC weakening and reversibility that can be directly related to CO_2_ emissions and global temperature changes.

One important caveat concerns the idealized nature of the experimental design. While the positive CO_2_ emission rate used during ramp-up (12.4 PgC/year; see Materials and Methods) is broadly comparable to the present level of real-world emissions, the negative emission rate applied during cooling is equal in magnitude and far exceeds what is considered feasible for carbon dioxide removal in realistic mitigation scenarios (*75*). However, sensitivity experiments with half the negative emission rate and ramp-downs started after 200 years of stabilization rather than 50 both show the same prompt AMOC recovery from 2- and 4-K GWLs (fig. S5). This demonstrates that the result is not sensitive to the specific cooling rate or the duration of the weakened state within the ranges tested. Whether this robustness extends to the slower carbon dioxide removal rates in realistic mitigation pathways remains to be tested.

Our conclusions rely on a single model (EC-Earth3-ESM-1). Multimodel analyses within the same TIPMIP ESM framework (*46*) will be essential for assessing the robustness of these results. Nevertheless, the simulated AMOC weakening and recovery behavior of our model can be compared to the broader CMIP6 ensemble. EC-Earth3-ESM-1 and its predecessor EC-Earth3 (which is a CMIP6 version of EC-Earth) (*76*) share the same atmospheric and oceanic components and tuning parameters (see Materials and Methods). In multimodel NAHosMIP, while some models reach a “collapsed” state and do not recover after freshwater hosing is ceased, a number of models—among them EC-Earth3—show less weakening without reaching a “collapsed” state and recover promptly (*34*), consistent with the reversible AMOC behavior found in our study. However, under global warming, EC-Earth3 falls near the middle of CMIP6 models in terms of AMOC decline under SSP2-4.5 scenario (*14*) and SSP5-8.5 scenario (*77*), indicating that the model is not systematically “stable” as the NAHosMIP intercomparison alone might imply.

If these results offer any reassurance, it is the gradual nature of AMOC weakening and its reversibility (i.e., without tipping)—both holding despite continued GrIS meltwater, an often assumed destabilizing forcing. AMOC reversibility should not, however, be mistaken for a lack of climate system impacts. (i) Temporally, recovery can unfold over centuries—far beyond human timescales—so the consequences of a weakened AMOC would persist over that same timescale, even though the AMOC does not tip. The recovery process itself may also be disruptive, although this was not assessed here: Some studies show regional temperature fluctuations that have knock-on effects for sea ice, permafrost, and ecosystems (*35*). (ii) Spatially, reversibility of AMOC strength—a single integrated volume transport metric for a far more complex system—does not imply that the associated wider North Atlantic system is equally reversible: The spatial pattern of MLDs after recovery differs from the original state (fig. S3), and to what extent related properties such as sea surface temperature and salinity recover remains to be assessed. (iii) Achieving recovery in practice, in any case, depends on the feasibility of the negative-emission rates discussed above.

Together, these considerations argue that avoiding an AMOC decline through rapid emission cuts in the first place remains a far safer strategy, rather than relying on recovery. The case is even stronger for the GrIS, which has no comparable recovery pathway: Delayed mitigation commits it to substantial and effectively irreversible mass loss and long-term sea level rise.

## MATERIALS AND METHODS

### Model and experimental design

We use EC-Earth3-ESM-1, an updated, post-CMIP6 version of the EC-Earth3 model (*76*), which couples atmosphere, ocean, sea ice, land, dynamic vegetation, ocean biogeochemistry, and a prognostic atmospheric CO_2_ box model, enabling simulations in emission-driven mode. Compared to the EC-Earth3 configuration, EC-Earth3-ESM-1 adds a fully interactive GrIS via the Parallel Ice Sheet Model (PISM) (*78*), a freshwater emulator for the Antarctic ice sheet (*79*), and an updated version of the LPJ-GUESS dynamic vegetation model (*80*); the atmospheric and oceanic components and their tuning parameters are unchanged relative to EC-Earth3. The GrIS coupling in EC-Earth3-ESM-1 is an updated implementation that closely follows previous EC-Earth3 configurations (*66*, *76*): PISM receives downscaled surface mass balance and temperature fields from the atmospheric and ocean modules to compute dynamically driven elevation changes and time-varying freshwater fluxes, which are fed back directly to the atmosphere and ocean.

Our simulations follow and extend the ESM protocol of the TIPMIP (*45*, *46*). Each model is forced by a constant CO_2_ emission rate that produces a global mean warming rate of 0.2 K/decade based on its transient climate response to cumulative CO_2_ emissions. For EC-Earth3-ESM-1, this corresponds to 12.4 PgC/year.

Simulations include the following:

1) A 1000-year PI control after spin-up;

2) A historical run (1850–2014);

3) Five ramp-up ensemble members with positive emissions, starting from PI states, each reaching ∼7-K global warming;

4) One extended ramp-up simulation (r1) continuing to ∼10-K warming;

5) Zero-emission simulations starting from GWLs of 1.5, 2, 3, 4, 5, 6, 7, 8, and 9 K, each branched from the first ramp-up member (r1) and run for 300 years (1.5 to 6 K) or 50 years (7 to 9 K);

6) Ramp-down simulations branched after 50 years of stabilization, using negative emissions at the same rate as during ramp-up, continued until GMSAT returns to the PI level (1.5 to 7 K) or for 250 and 150 years for the 8- and 9-K cases, respectively;

7) Two sensitivity ramp-up simulations with doubled and tripled emission rates, both reaching ∼7-K global warming, to assess the potential impact of forcing rate–induced AMOC change;

8) Four sensitivity ramp-down simulations to assess the sensitivity of AMOC reversibility to the ramp-down pathways; we conducted 2- and 4-K GWL ramp-downs in which (i) negative emissions were applied at half the rate and (ii) the experiments were branched after 200 years of stabilization rather than 50.

We substantially extended the TIPMIP ESM tier 1 experiment design, which originally included only a ramp-up to 4 K, zero-emission simulations starting from 2 and 4 K, ramp-down simulations from 2 and 4 K down to PI temperature levels, and another zero-emission simulation when the 4-K ramp-down reaches 2 K (*46*). The 8- and 9-K ramp-down simulations do not extend to PI temperatures due to computational limits, but both show clear AMOC recovery, which is expected to continue and eventually overshoot the PI strength.

All experiments are emission driven: Atmospheric CO_2_ concentration evolves prognostically (fig. S6), allowing coupled carbon–climate feedbacks to influence global temperature and thus ocean circulation and ice sheet responses. This framework and setup enable direct assessment of transient AMOC and GrIS reversibility under comparable warming and cooling trajectories.

### Calculations of AMOC

AMOC strength, as used throughout Results and Discussion, is calculated as the overturning stream function at 26°N at the fixed depth of 730 m, which corresponds to the depth of the maximum stream function in the PI control simulation, following Drijfhout *et al.* (*19*). Using a fixed depth can underestimate the AMOC strength when the upper overturning cell shoals under global warming, and using a fixed latitude cannot capture the true latitudinal maximum nor any shift in its position under global warming.

We test the sensitivity of our results to both choices using dynamically tracked alternatives (fig. S4): the vertical maximum of the stream function at 26°N, excluding the upper 200 m to remove the wind-driven component (fig. S4B), which relaxes the depth constraint; and the same approach applied across a wider latitudinal range (between 20°N and 50°N; fig. S4C), which relaxes both the depth and latitude constraints. Since the fixed-depth metric increasingly underestimates the true maximum as the overturning shoals, allowing depth to vary yields a stronger AMOC strength than the fixed-depth metric (∼5 Sv versus ∼3 Sv at the weakest value); searching across a wider latitudinal range yields a further increase of 1.5 Sv.

To facilitate comparison with NAHosMIP (*34*), the AMOC strength is also calculated as the vertical maximum at 45°N (fig. S4D). All four metrics yield qualitatively similar AMOC behavior.

### Calculations of MLD

The MLD diagnostic in this study is defined as the depth where the potential density referenced to the surface increases by 0.01 kg/m^3^ relative to 10 m depth. As there is no single depth level threshold that defines deep-water formation (*81**,* *82*), we identify potential deep-convection regions as those where the mean March MLD exceeds 650 m in the PI control simulation—the same threshold used by Drijfhout *et al.* (*19*), although applied there to the period of 1965–1995 in the historical simulations. We use the long PI control specifically to avoid the large internal variability evident in these regions (Fig. 3). These fixed deep-convection regions (shown as black contours in Fig. 2) are separated into three subregions: the Labrador, Irminger, and Nordic Seas—where the Labrador and Irminger Seas are divided at the southern tip of Greenland (59°N). To quantify temporal variability, we extract March MLDs within each subregion and compute spatially weighted means.
