# Supplementary material for: Reversible Atlantic overturning despite continued Greenland ice sheet melt
Source: Sci Adv. 2026 Oct 14;12(42):eaee5373. doi: 10.1126/sciadv.aee5373 (PMC13665990; doi:10.1126/sciadv.aee5373)
Supplement: Supplementary file 1 [file sciadv.aee5373_sm.pdf]

Science Advances
AAAS

Supplementary Materials for

Reversible Atlantic overturning despite continued Greenland ice sheet melt

Chuncheng Guo *et al.*

Corresponding author: Chuncheng Guo, chc@dmi.dk

Sci. Adv. **12**, eaee5373 (2026)
DOI: 10.1126/sciadv.aee5373

This PDF file includes:

Figs. S1 to S6

Supplementary figures

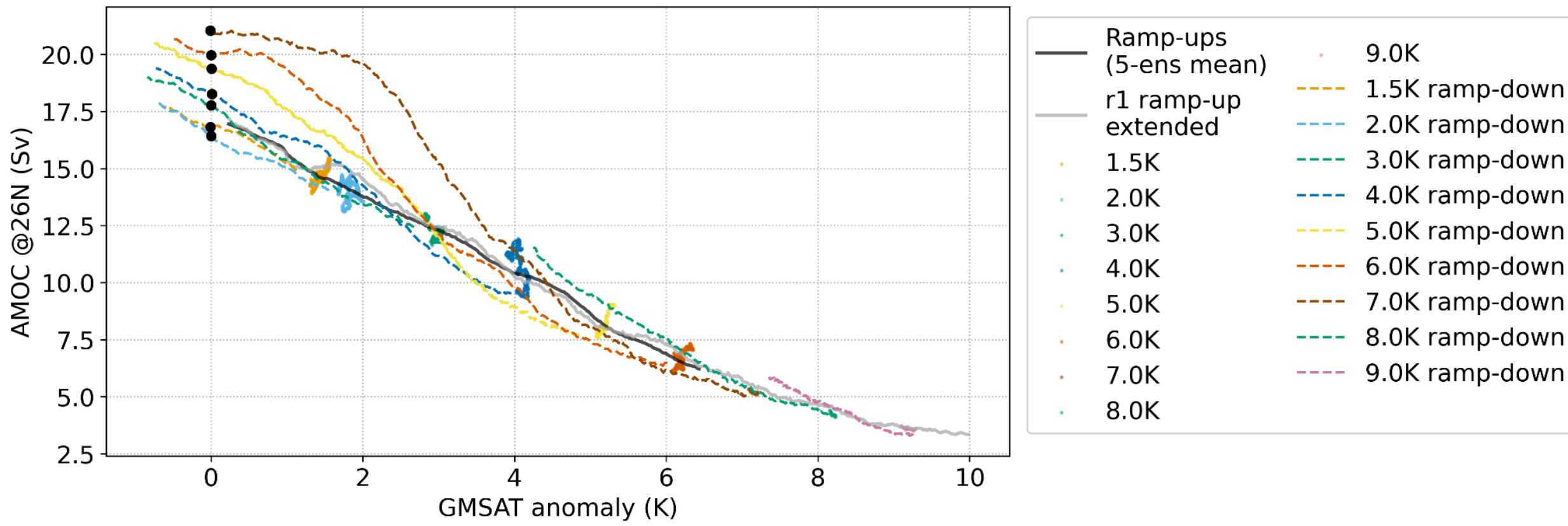

Supplementary Fig. 1: Sensitivity of AMOC strength to global mean surface air temperature.

AMOC strength at 26°N (defined at the depth of the PI maximum, 730 m) as a function of the global mean surface air temperature (GMSAT) anomaly for the ramp-up (warming), stabilisation, and ramp-down (cooling) simulations. The black line shows the 5-member ensemble mean from the ramp-up runs; the grey line indicates one extended ramp-up run. The scattered dots denote the individual global warming levels from 1.5-9 K, and dashed lines show the corresponding ramp-down simulations. Black dots mark when the GMSATs return to the PI level. The nearly overlapping trajectories demonstrate a prompt recovery of the AMOC, while ramp-downs from higher warming levels exhibit stronger transient overshoots upon recovery.

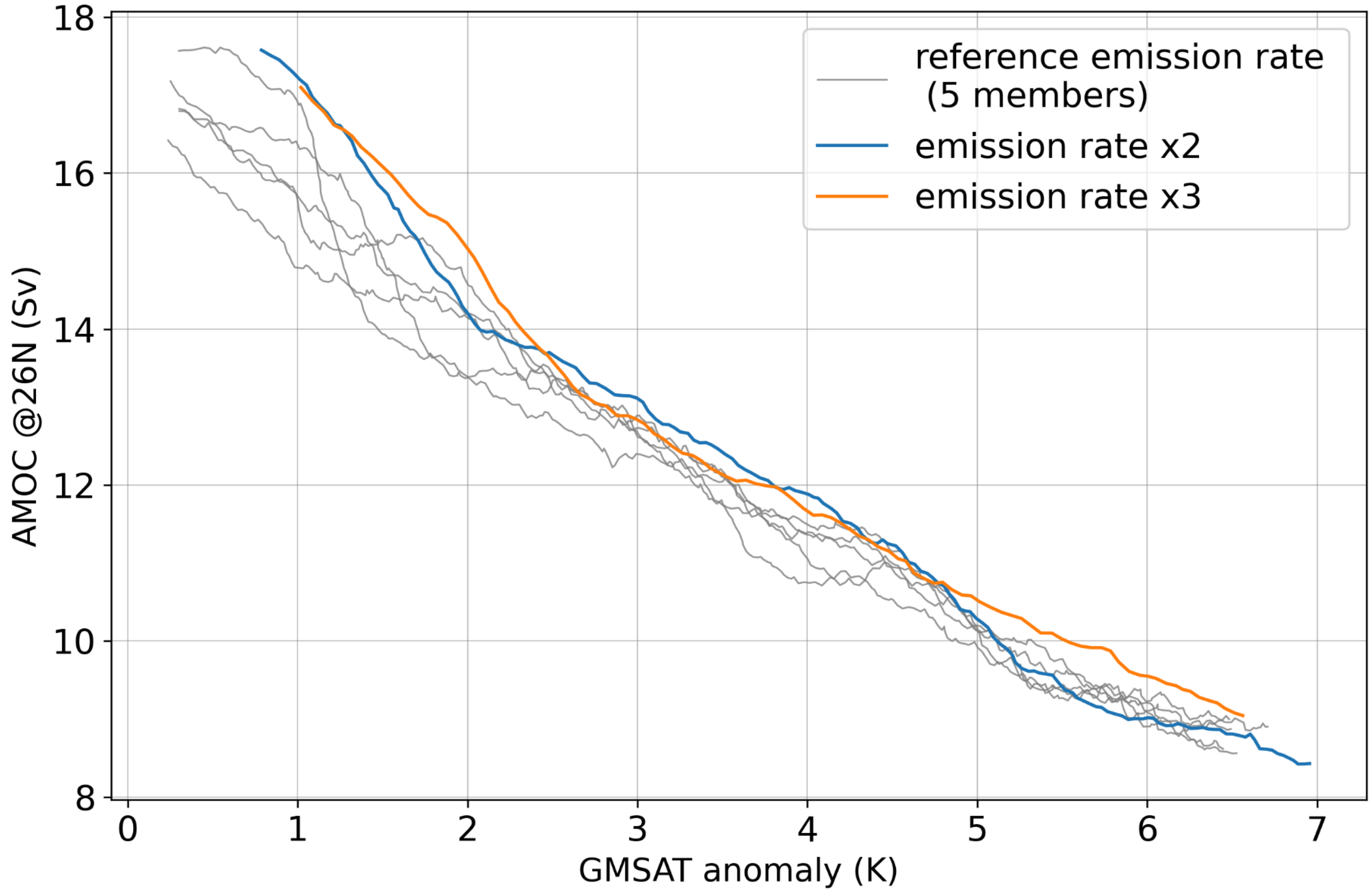

Supplementary Fig. 2: Sensitivity of AMOC response to CO_2 emission rate in the ramp-up experiments.

AMOC strength at 26°N as a function of global mean surface air temperature (GMSAT) anomalies for the ramp-up experiments. The grey lines show the five-member ensemble with the reference CO_2 emission rate (12.4 PgC/year), while blue and orange lines show additional sensitivity runs with doubled and tripled emission rates, which correspond to a warming rate of 0.37 and 0.54 K/decade, respectively. Despite the more rapid forcing, the AMOC trajectories converge onto a near-single curve when plotted against GMSAT, indicating that in EC-Earth3-ESM-1 the AMOC response scales primarily with the level of warming rather than its rate within the forcing range tested here.

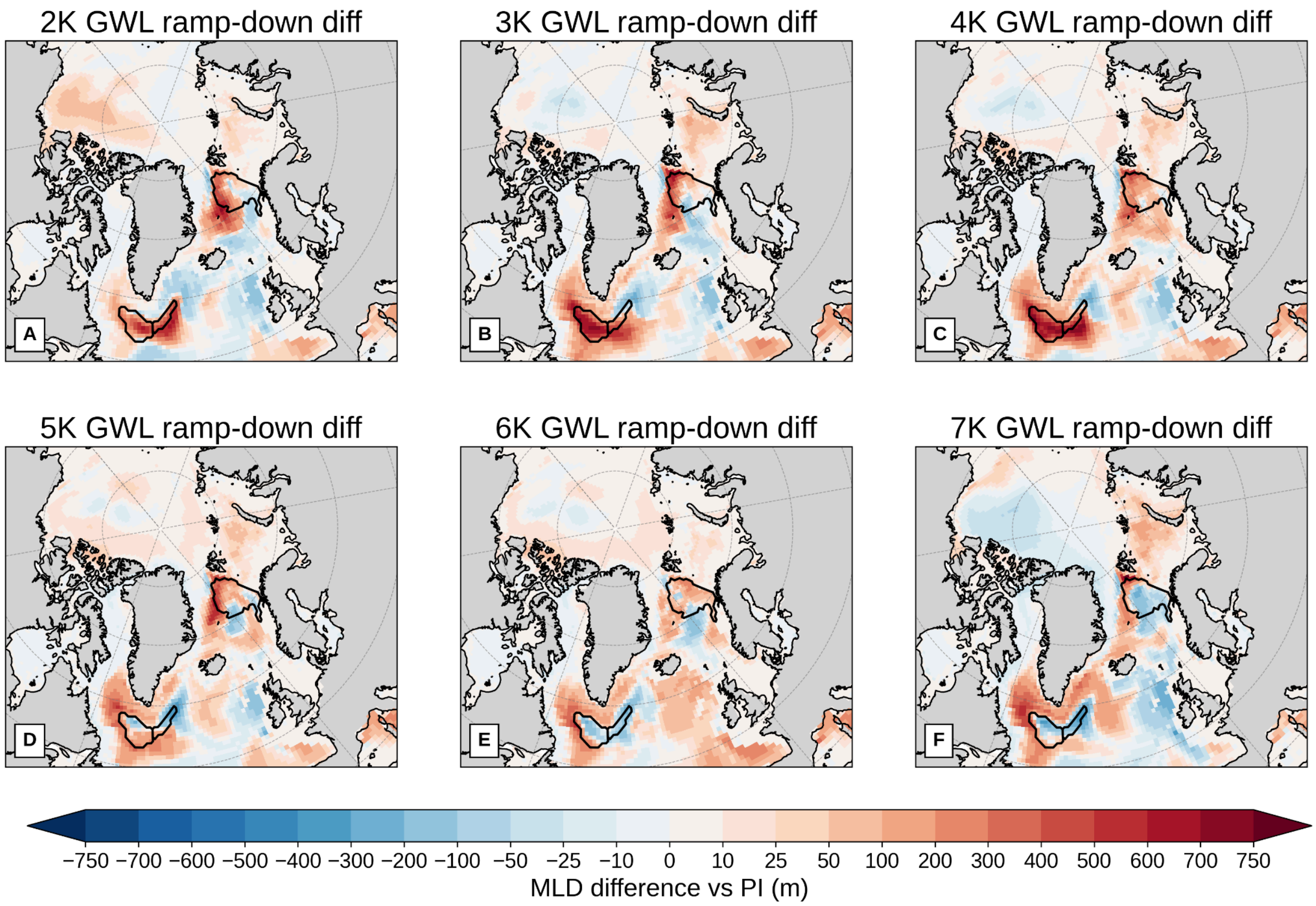

Supplementary Fig. 3: Spatial differences in March mixed-layer depth during ramp-down simulations returning to pre-industrial temperatures.

(A-F) March mean mixed-layer depth (MLD) anomalies during the 2-7 K ramp-down phases, shown as differences relative to the pre-industrial MLD in Fig. 2A. Each panel represents a 30-year mean centred on the year when the global mean temperature returns to the pre-industrial level. The black contours mark areas where the pre-industrial MLD exceeds 650 m, indicating the main deep convection sites in the Labrador, Irminger, and Nordic Seas. The maps show that deep convection areas reorganise spatially during recovery; MLD patterns are not identical to PI even when AMOC strength has recovered. These results are based on a single realisation of the model and therefore include contributions from internal variability.

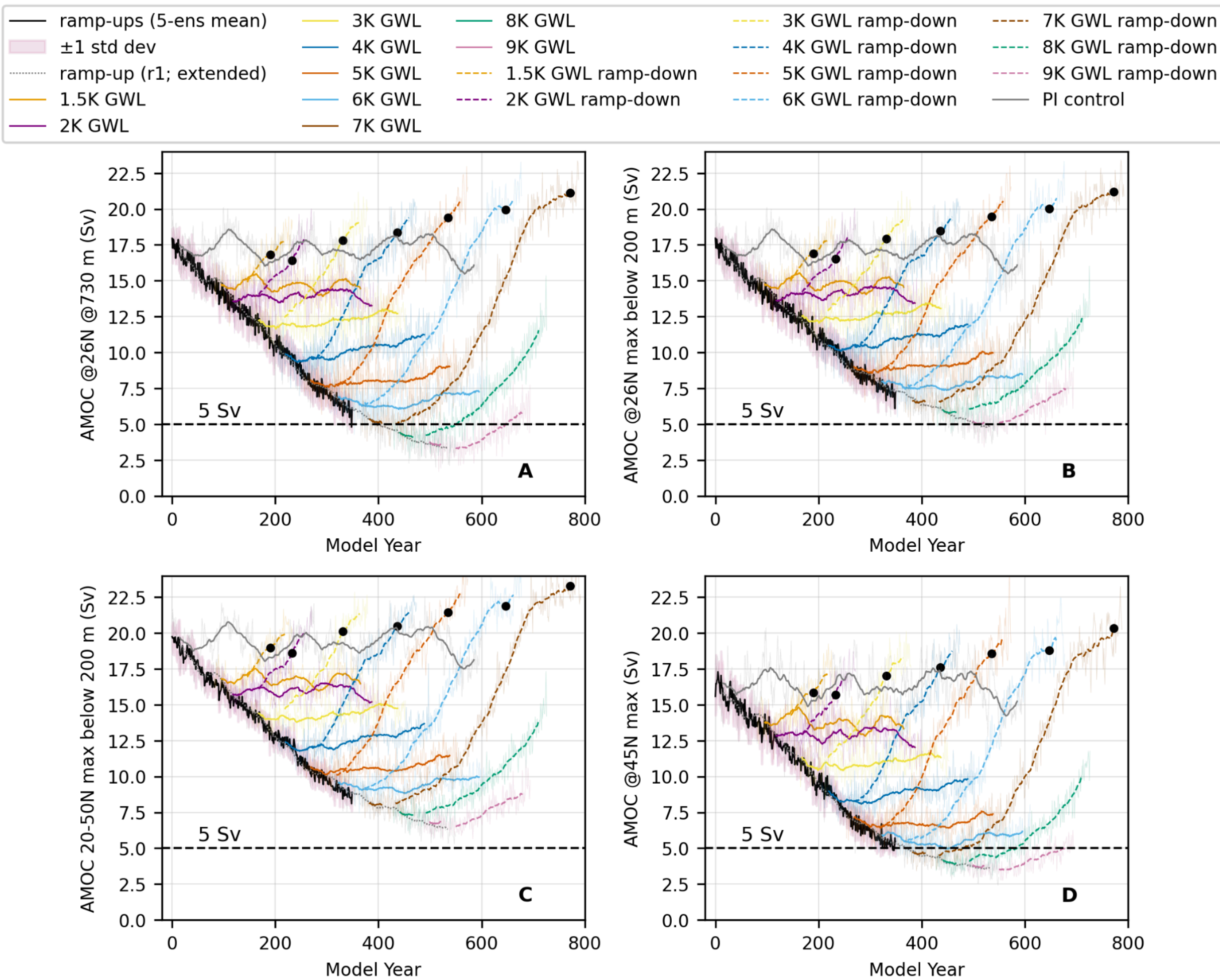

Supplementary Fig. 4: Sensitivity of simulated AMOC strength to the choice of definitions under idealised CO_2 emission scenarios.

AMOC strength calculated using four different definitions: **(A)** fixed depth at 26°N (730 m; as used throughout the main text and in Fig. 1B), **(B)** the vertical maximum below 200 m at 26°N, excluding the wind-driven surface cell, **(C)** the vertical maximum below 200 m between 20°N and 50°N, and **(D)** the vertical maximum at 45°N. A horizontal reference line at 5 Sv shown in each panel is to aid visual comparison when each definition indicates a weak AMOC state.

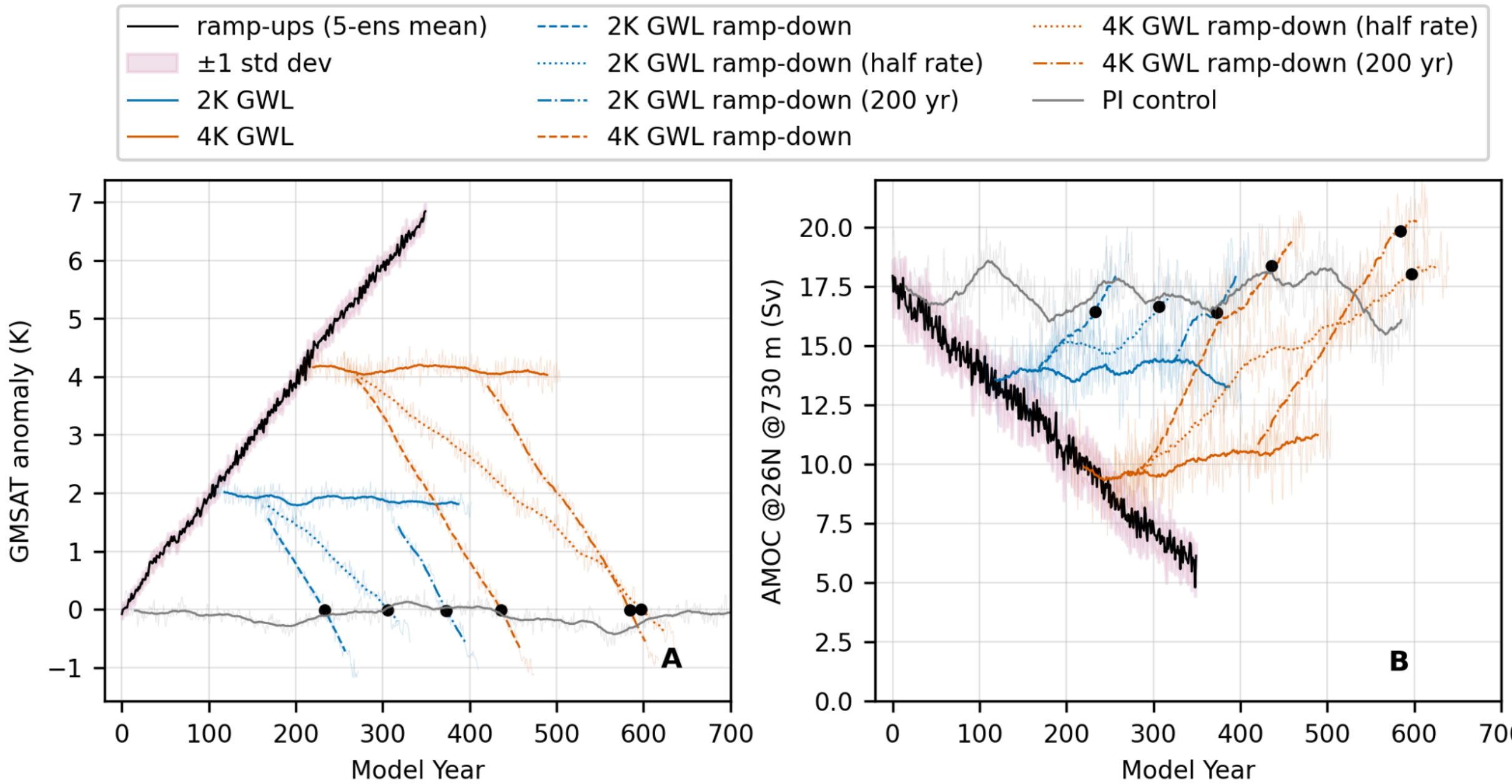

Supplementary Fig. 5: Same as Fig. 1, but for the sensitivity experiments of 2 K and 4 K GWL ramp-downs. The reference ramp-down applies negative emissions at the same rate as the ramp-up; the "half rate" variant applies negative emissions at half that rate; the "200 yr" variant denotes the ramp-down starting after 200 years of stabilisation rather than 50.

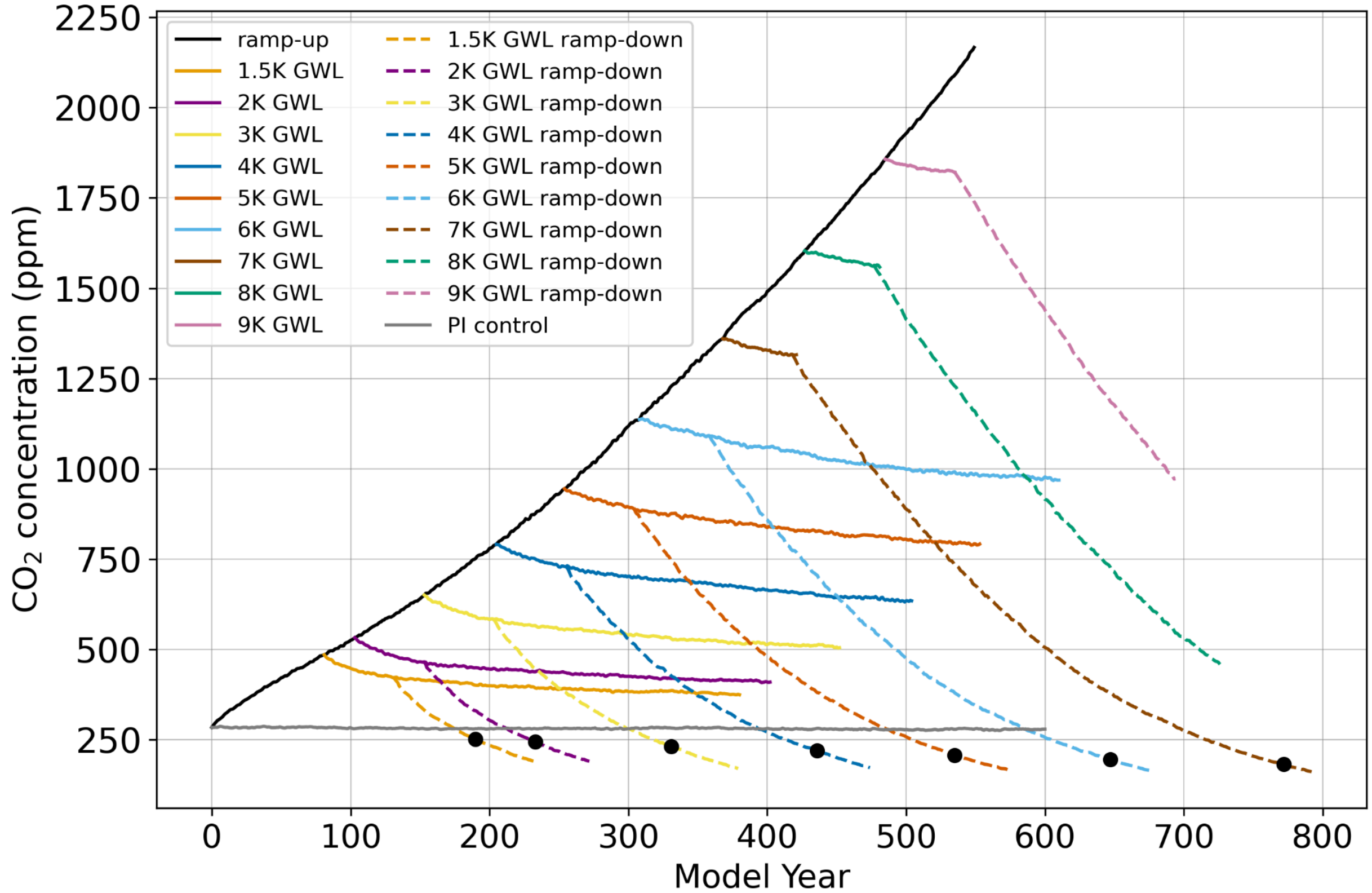

Supplementary Fig. 6: Atmospheric CO_2 concentrations under idealised CO_2 emission scenarios.

Evolution of global mean atmospheric CO_2 concentration (parts per million; ppm) in the ramp-up (positive emissions), stabilisation (zero emissions), and ramp-down (negative emissions) experiments with EC-Earth3-ESM-1. Colours and line styles correspond to those in Fig. 1. Black dots mark when the GMSATs return to the pre-industrial level in the ramp-down experiments. CO_2 concentration is stable in the PI control simulation, and closely follows the observations in the historical period. The concentration pathways confirm the model's emission-driven configuration, linking imposed emissions directly to the transient temperature responses shown in Fig. 1A.
